# Inhibition of Galectin-3 Impairs Antifungal Immune Response in Fungal Keratitis

**DOI:** 10.1155/2022/8316004

**Published:** 2022-04-09

**Authors:** Yichen Xiao, Jiahui Yang, Zhenyuan Fu, Zhile Xiong, Chao Zhang, Dalian He, Zhenwen Zhou, Naiyang Li, Jin Yuan

**Affiliations:** ^1^State Key Laboratory of Ophthalmology, Zhongshan Ophthalmic Center, Sun Yat-sen University, Guangdong Provincial Key Laboratory of Ophthalmology and Visual Science, Guangzhou 510060, China; ^2^Clinical Laboratory, Guangzhou Women and Children's Medical Center, Guangzhou Medical University, Guangzhou, Guangdong 510623, China; ^3^Eye Center, Zhongshan Hospital of Sun Yat-sen University, Zhongshan, Guangdong, China

## Abstract

Galectin-3 is one of the galectin family members which are master regulators of immune homeostasis, especially in infectious diseases. However, its mechanism of immune regulation in fungal keratitis has not been thoroughly studied. Our study is aimed at clarifying the role of galectin-3 in the fungal keratitis mouse model in vivo, thereby providing a new biomarker of antifungal therapy. In our study, aspergillus, the most common pathogenic fungi of fungal keratitis, was identified and isolated by corneal tissue fungus culture. Then, the RNA expression levels of galectin family members in corneas of the mouse model with aspergillus fumigatus keratitis were screened by transcriptome sequencing (RNA-seq). The expression of the galectin-3 was detected by quantitative real-time Polymerase Chain Reaction (qPCR), enzyme-linked immunosorbent assay (ELISA), and immunofluorescence in the corneal tissue of the fungal keratitis mouse model. Recruitment of neutrophils and the co-immunofluorescence of galectin-3 and related markers in corneal tissue were determined by flow cytometry analysis and immunofluorescence staining. The regulatory role of galectin-3 for proinflammatory cytokines and neutrophils was validated by the knockout mouse model. Galectin-3 knockout deteriorated the condition for the inhibition of galectin-3 was benefecial for fungi to survive and thrive in corneal lesions. These results demonstrated that in the ocular fungal infection, galectin-3 is capable of regulating the pathogenesis of fungal keratitis by modulating neutrophil recruitment. The deterioration of fungal keratitis and increased fungal load in corneal lesions of galectin-3 knockout mice proved the regulatory role of galectin-3 in fungal keratitis. In conclusion, galectin-3 is going to be an essential target to modulate neutrophil recruitment and its related antifungal immune response in fungal keratitis.

## 1. Introduction

The human body, an intricate system protecting us from microbial attacks and invasions. Microbes live on/inside our body and play key roles in human physiology turbulence, and immune disorders [1]. Fungal keratitis (FK), one of the most detrimental ocular diseases caused by a fungal infection, loomed so large to be a key health issue of great importance [2, 3]. There are at least 100,000 known species of fungi. However, fewer than 500 have been proved to cause disease in animals, including human beings. The incidence rate of aspergillus is the highest among the pathogenic fungi, according to epidemiologic studies [4–6]. The immunological mechanism of microbial infectious disease includes both pathogen clearance and anti-infection immune response [7]. The microbial ocular disease has been of great interest to us for over a decade [8, 9].

Galectins as a family are characterized by a galactose-specific carbohydrate-binding domain interacting with galactose moieties located at glycoproteins on the surface of the lipid bilayer of cell membranes [10–12]. The galectin group consists of 15 different galectins which have already been identified structurally and functionally [12–14]. The members of this family play different roles in immune homeostasis regulation [15]. Galectin-1 triggers homeostatic signals to turn off T-cell effector functions, galectin-9 promotes pro-inflammatory cytokines release by the signal of Toll-like receptors down-regulation, and galectin-7 and galectin-12 have effects on apoptosis. Galectin-3 always behaves as the amplifier of the inflammatory cascade, thereby playing a key role in inflammatory diseases [16–19]. Galectin-3, the only member containing carbohydrate recognition domain with an extended N-terminus, behaves as a regulator to amplify the process of the inflammatory cascade [20]. Accumulating evidence showed that galectin-3 is instrumental to many biological and pathobiological functions. Galectin-3 could regulate immune responses in infectious diseases such as fungal nephropathy, streptococcus pneumonia, and HSC virus infection by controlling immune cell activation, recruitment, and differentiation [15, 21–27].

It is worth noting that galectin-3 plays a central role in innate immune cell homeostasis, especially for neutrophils, which are the primary inflammatory cells during the disease course of fungal keratit [28]. Galectin-3 enhances the adherence between neutrophils and monolayers consisting of endothelial cells as well as the matrix proteins and fibronectins in vitro [29, 30]. The role of galectin-3 in immune cell regulation remains to be unveiled which contributes most to the antipathogen immune response in infectious diseases, especially the regulation of neutrophils. The current accumulation of new information depicts a future scenario where galectin-3 could be used as a potential anti-inflammatory mediator and a specific modulator of the immune response in inflammatory and infectious disease, possibly by regulating neutrophils and other related immune responses. However, its role in regulating ocular fungal infection remains unknown.

This study aims to explore potential target and the underlying mechanism of fungal keratitis caused by the typical strain of pathogenic fungi with high incidence. The fungal infection mouse model would be established, and the correlation between key immune cells and biomarkers would be explored. This study is designed and aimed at providing evidence to support the vital role of galectin-3 in fungal keratitis immune disorders, pointing out the key disease marker. Targeting galectin-3 for antifungal therapy based on the emerging findings of biology and pathology related to galectin-3 is coming of age. Therefore, it is going to shed some light on patients with fungal keratitis all over the world.

## 2. Methods

### 2.1. Patient and Tissue Specimens

The tissue samples, ocular image, periodic acid-Schiff Stain (PAS), and microbial culture results were collected from patients who were clinically diagnosed with fungal keratitis by corneal scraping culture and received corneal transplantation from May 2020 to May 2021 at the Zhongshan Ophthalmic Center. This study was approved by the Zhongshan Ophthalmic Center Medical Science Research Ethics Committee (protocol number: 2020KYPJ115). All participants in this study provided written consent. The infected corneal tissues were collected during corneal transplantation surgery and quickly stored in a cryogenic refrigerator at -80°C.

### 2.2. Preparation of Aspergillus Fumigatus Spores

The typical fungal strain was cultured on Potato dextrose agar (PDA) or Sabouraud dextrose agar (SDA, Difco, Detroit, Michigan, USA) at 30°C. The presence of fungal stain in the corneas of patients was confirmed by the characteristic branching hyphae and granular spores of Aspergillus under the microscope. The strain of Aspergillus fumigatus used in this investigation was AS 3.1320,purchased from the Guang Dong Microbiological Culture Collection Center, Gouangzhou, China. The Aspergillus fumigatus strain was grown on Sabouraud dextrose agar (Difco, Detroit, MI) at 30°C for 4 days. After washing the dishes in phosphate-buffered saline with 0.1% Tween 20 (PBST), the spore suspension was harvested by washing in sterile phosphate-buffered saline (PBS). All spore solutions were diluted to a 5 × 10^5^ colony-forming units (CFU)/mL concentration with a cell counting chamber. The preparation of Aspergillus fumigatus spores was completed in the Laboratory of Guangzhou Women and Children's Medical Centre (Guangzhou, China).

### 2.3. Experimental Animals

150 female C57BL/6N mice (6-8 weeks old), weighing 18-21 g, were purchased from Beijing Vital River Laboratory Animal Technology Co. Ltd., and the construction of galectin-3 complete knockout mice was commissioned by Saiye Biology (Strain name: C57BL/6N-Lgals3em1cyagen, strain number: KOCMP-16854-Lgals3-B6N-VA). The mice were divided into four groups: wild type group, wild type fungal keratitis group, galectin-3^−/−^ group, and galectin-3^−/−^ fungal keratitis group. Animal breeding and experiments were carried out in the Specific Pathogen-Free (SPF) animal room of the Experimental Animal Center of Sun Yat-sen University Zhongshan Ophthalmic Center. All animals were treated following the guidelines provided by the Ophthalmology and Vision Research Animal Use Vision and Ophthalmology Research Association and were approved by the Zhongshan Ophthalmic Center Institutional Review Committee (ethics number: 2020-011). All laboratory animal use followed the Association for Research in Vision and Ophthalmology (ARVO) requirements.

### 2.4. Animal Model In Vivo

To establish the fungal keratitis model in vivo, C57BL/6N mice or galectin-3^−/−^ mice were anesthetized intraperitoneally with sodium pentobarbital (1%) and lidocaine hydrochloride (0.5%) was applied to the eye surface twice or more for corneal anesthesia. First, a 30-gauge needle was used to form a tunnel in the corneal stroma of the right eye. Then, we used a 33-gauge syringe to puncture through the tunnel and injected 5 *μ*L spore solution (5 × 10^5^CFU/mL) into the corneal stroma until the corneal stroma turned into a uniform white color. The untreated left eye of each mouse was regarded as a control for the disease course of fungal keratitis. After the mice were sacrificed with an overdose of anesthesia, the corneal tissues were harvested.

To illustrate disease progression, mouse corneas were photographed to determine clinical scores at 5 days postinfection (dpi). The severity of fungal keratitis was graded on a scale ranging from 0 to 12 according to a scoring system developed by Wu et al. [31]. The clinical scores were calculated by three aspects: area of corneal opacity (0-4), density of corneal opacity (0-4), and corneal surface regularity (0-4).

### 2.5. RNA-seq and Data Analysis

RNA samples were extracted from corneas of the mouse model with fungal keratitis at 5 dpi (*n* = 3/group/time point) following the procedure as instructed by RNeasy Plus Mini Kit (Qiagen, Valencia, CA, USA). RNA-seq library construction and computer sequencing were completed by Annoyoda Biotechnology Co. Ltd., and trimmomatic software was applied to remove the adapter sequence from the original data, low-quality bases, and low-quality reads. We used Hisat2 software to compare valid reads to the human GRCh38 reference genome and RSEM software to calculate the TPM value of each sample. R package DESeq2 was used to analyze differentially expressed genes. Differential gene expression was defined as log2-fold change > 1 and *p* value < 0.05. R package cluster profiler was used to perform GO BP and KEGG enrichment analysis for differential genes. *p* value cutoff of 0.01 and *q* value cutoff of 0.05 were thresholds to filter significantly enriched GO terms.

### 2.6. Quantitative Real-Time Polymerase Chain Reaction (qRT-PCR)

To examine galectin-3 mRNA expression in fungal keratitis mice and compare the expression of C-X-C Motif Chemokine Ligand 1 (CXCL1) and Interleukin-1*β* (IL-1*β*) mRNA between wild type and fungal keratitis mice, fungal keratitis mice, and Galectin-3 knock-out fungal keratitis mice, we first used Qiagen RNA extraction kit to extract total RNA from the corneas of fungal keratitis mice, according to the instructions and TaKaRa Reverse Transcription Kit to perform RNA reverse transcription in a PCR machine. Transcripts were quantified via SYBR Green qPCR performed an iQ Thermocycler (Bio-Rad) or ABI using Quant Studio Design v1.3. Relative expression of RNA was normalized, and the data were analyzed using the 2−*ΔΔ*Ct method. All qRT-PCR experiments were repeated three times. The primers were purchased from Invitrogen, and their sequences are shown as follows:

1. Galectin-3: forward, 5′-GCTTATCCTGGCCCAACTGC-3′; reverse: 5′-CCCCGCTGGACCACTGACGG-3′
2. IL-1*β*: forward, 5′-TGTCGGACCCATATGAGCTG-3′; reverse: 5′-TCCTTTGAGGCCCAAGGCCA-3′
3. CXCL-1: forward, 5′-CAAACCGAAGTCATAGCCAC-3′; reverse, 5′-TGGGGACACCTTTTAGCATC-3′
4. GAPDH: forward, 5′-CTCATGACCACAGTCCATGC-3′; reverse, 5′-TTCAGCTCTGGGATGACCTT-3′

### 2.7. Enzyme-Linked Immunosorbent Assay (ELISA)

The protein levels of galectin-3 and CXCL1 were selectively tested using ELISA kits (R&D Systems). Corneal samples of fungal keratitis mice were individually collected at 5 dpi and homogenized in 0.5 mL PBS containing 0.1% Tween-20. All samples were centrifuged for 5 minutes, and then, the supernatant was assayed under the manufacturer's instructions.

### 2.8. Flow Cytometry Analysis

To determine the proportion of neutrophils in different stages of fungal keratitis mice, infected corneas were harvested at 5 dpi and cut into pieces (about 1 mm^3^) under the microscope. According to the manufacturer's instructions, each tube was added with 60 U/mL Liberase TL (Roche Diagnostics, USA) and 1 mL serum-free 1640 medium and placed in a 37°C incubator for lysis for about 45 minutes, shaking several times every 15 minutes. The digested tissue and its suspension were filtered with a 70 *μ*m nylon mesh to form a single cell suspension in 10% 1640 medium for staining. After that, cells were blocked with unconjugated anti-CD32/anti-CD16 mAbs for 15 min in FACS buffer (2% fetal bovine serum in PBS). Next, cells were treated with FITC-conjugated CD45 antibody, PE-conjugated Ly6G antibody, and BV 421-conjugated CD11b antibody for 30 min on ice. The cells were then resuspended in flow cytometry staining buffer and analyzed in a Cyto-FLEX S flow cytometer (Beckman, USA).

### 2.9. Immunofluorescence Staining

To detect the expression of Cytokeratin 12 (CK12), galectin-3, and CD11b in fungal keratitis, immunofluorescence staining in mouse corneas of fungal keratitis was performed. Corneal tissues were fixed with 4% paraformaldehyde for 10 minutes. The mouse cornea tissues were then equilibrated in 30% sucrose solution overnight for cryoprotection, embedded in OCT media, and sectioned using a Cryostat set at 10 *μ*m per section. Slides of corneal tissues were saturated with 0.5% Triton X-100 in PBS for 60 minutes and blocked with 3% BSA for 30 minutes. The slides and cells were incubated with primary antibodies specific for CK12 (1 : 200, Abcam, UK), galectin-3 (1 : 200, Abcam, UK), and CD11b (1 : 500, Abcam, UK) for one hour at room temperature. After washing 5 minutes, 3 times in PBS, slides were incubated with respective secondary antibodies of Alexa Fluor 594-conjugated IgG (1 : 300, Abcam, UK) or Alexa Fluor 488-conjugated IgG (1 : 300, Abcam, UK) for one hour at room temperature, and the nucleus was stained with 4′,6-diamidino-2-phenylindole (DAPI) for 10 minutes. Fluorescence images were acquired by a laser confocal fluorescence microscope (LSM800; Carl Zeiss Microscopy, White Plains, NY, USA).

### 2.10. Hematoxylin-Eosin (HE) Staining

To determine the pathological changes of fungal keratitis, the entire eyeballs of fungal keratitis mice (*n* = 3/group/time point) were harvested at 5 dpi, fixed with 4% formaldehyde overnight. Then, the corneal tissues were dehydrated through a graded ethanol series (70–99%), embedded in paraffin, cut into sections (5 *μ*m thick), and stained with HE.

### 2.11. Two-Photon Microscopy (TPM)

To detect the existence of hyphae in vivo, we performed cornea imaging of both FK mice and galectin-3 knockout FK mice using a custom-built TPM system. Corneal scraping was performed to obtain the corneal sample for imaging. TPM used a Ti-Sapphire femtosecond laser (Chameleon Ultra II; Coherent, Inc., Santa Clara, CA) as a light source. A ×20 objective lens [XLUMPlanFLN, ×20, 1.0 numerical aperture (NA), water immersion; Olympus] was used. The imaging field of view and imaging speed was approximately 300 × 300 *μ*m in the transverse *x*-*y* plane consisting of 512 × 512 pixels and 0.1 frame/s, respectively. The excitation wavelength was 780 nm for both intrinsic contrast and moxifloxacin-based imaging. Autofluorescence and SHG signals were collected in two detection channels by separating emission light with a 430 long-pass dichroic mirror. Then, ImageJ was used to form the color fungal hypha image.

### 2.12. Fungal Load Counting

Infected mouse corneas (*n* = 3/group/time point) were harvested at 5 dpi, then washed, and homogenized in 1000 *μ*L of normal sterile saline. Serial dilutions were made accordingly to achieve a proper number of colonies on each plate. A total of 100 *μ*L of corneal tissue suspensions were dropped on respective PDA plates and evenly spread with a spreader. CFU counting was calculated after 18–24 h incubation at 37°C. Aspergillus fumigatus loads were expressed as CFU/mL. Petri dishes that were only exposed to the clean bench for 10 s were regarded as a negative control.

### 2.13. Statistical Methods

The results are presented as the mean ± standard error of the mean (SEM) values. Statistical analyses were performed using SPSS for Windows (version 22.0; International Business Machines Corporation). An independent *t*-test was used to compare the differences between the two groups. One-way analysis of variance (ANOVA) with Dunnett's test for multiple comparisons was performed to assess the significance of experimental groups versus the control group. The images were generated by GraphPad Prism 7 for Windows (version 7.04; GraphPad Software). *p* values less than 0.05 were considered significant.

## 3. Results

### 3.1. Aspergillus Was One of the Most Common Pathogenic Fungi of Fungal Keratitis

**Figure 1:**
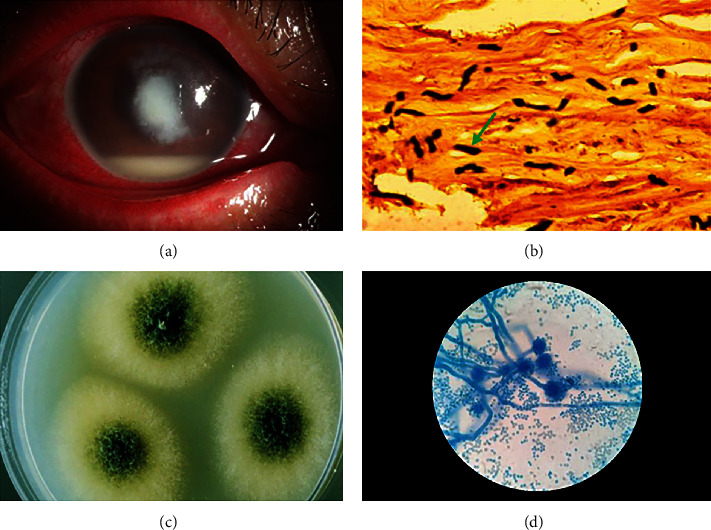
The cornea samples of the patients with fungal keratitis with the typical anterior segment photographic appearance (a) and corneal PAS staining results (b) were collected to culture fungi. Aspergillus fumigatus was isolated from the culture flora (c). It was lactic acid phenol cotton orchid stained to reveal the typical aspergillus morphology under the microscope, including branching hyphae and granular spores (d).

Aspergillus was one of the most common pathogenic fungi of fungal keratitis, and Figure 1(a) shows a representative fungal keratitis typical clinical signs like corneal inflammatory ulcer and corneal tissue PAS staining showing the presence of a large number of fungi (green arrow) in the stroma (Figures 1(a) and 1(b)). To detect the main pathogenic microorganism in the cornea of patients, the corneal tissue was extracted and cultured fungi. Then, the fungi with the highest proportion screened by the laboratory department were further cultured, which showed the characteristic cotton-like aspergillus appearance (Figure 1(c)). The lactic acid phenol cotton orchid staining of the fungi revealed dendritic hyphae and scattered granular spores under the microscope, proving typical characteristics of aspergillus (Figure 1(d)).

### 3.2. The Expression of Galectin-3 was Significantly Increased in the Corneal Lesions of the Fungal Keratitis Model

**Figure 2:**
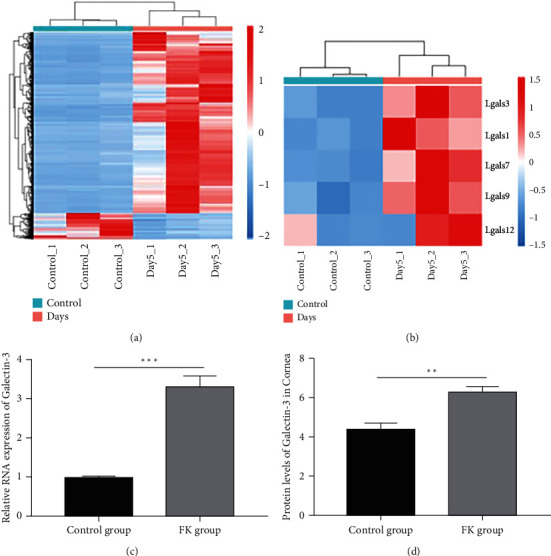
Analysis of RNA-seq in the cornea of a mouse model of fungal keratitis was used to screen the targets in the disease. (a) was the number of differential genes between the mode and the control groups. The log2-fold change sequencing screened out highly expressed genes in the galectin family, galectin-3 expressing obviously (b). Meanwhile, qPCR (c) results in mouse cornea or ELISA (d) in corneas verified the upregulated expression of galectin-3 in fungal keratitis. ^∗^*p* < 0.05;  ^∗∗^*p* < 0.01;  ^∗∗∗^*p* < 0.001; NS: no statistical difference.

RNA-seq sequencing analysis was performed in the extracted corneas of the mouse model to screen out the key target of fungal keratitis. Among the detected genes, 3025 upregulated and 2654 downregulated genes were found on the fifth day after being infected, which was the peak of the disease (Figure 2(a)). Meanwhile, the galectin family was significantly expressed in the upregulated gene spectrum, which aroused our attention, and we further analyzed them. The results showed that the expression of galectin-3 increased most significantly among the galectin family members. (Figure 2(b)). In addition, the RNA expression of galectin-3 (3.32 ± 0.26, *p* < 0.001) (Figure 2(c)) or the protein expression level of galectin-3 (Figure 2(d)) in corneas (6.40 ± 0.14 ng/mL, *p* < 0.01) also showed that galectin-3 was significantly increased in the disease model compared to the normal group. It suggested that galectin-3 may play an important role in fungal keratitis.

### 3.3. Galectin-3 and Neutrophils were Colocalized during the Disease Development of Fungal Keratitis

**Figure 3:**
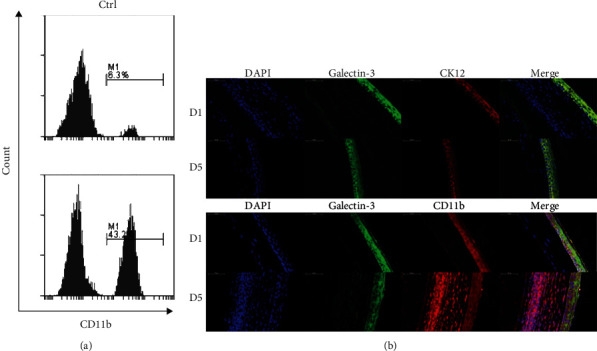
The cell frequency rate of neutrophil (a) detected by flow cytometry analysis at the 5 days post-infection. Immunofluorescence co-staining (b) of galectin-3 (green fluorescence) and cornea (CK12, red fluorescence) or neutrophil (CD11b, red fluorescence) indicated that galectin-3 and neutrophil activation were highly correlated at one day postinfection and 5 days postinfection. ^∗^*p* < 0.05;  ^∗∗^*p* < 0.01;  ^∗∗∗^*p* < 0.001; NS: no statistical difference.

The mouse model of fungal keratitis was established to further verify the high expression of galectin-3 in fungal keratitis and its potential regulatory mechanism. We calculated the changes in the levels of neutrophil activation during disease by flow cytometry analysis.  Figure 3(a) shows that neutrophils (CD45+ ly6G+ CD11b+) were activated obviously during the course of the disease (37.80 ± 1.87%, *p* < 0.001) in corneas, indicating neutrophils to be the primary functional cell subtype in antifungal immune responses. Meanwhile, the results of immunofluorescence showed that galectin-3 (green fluorescence) was coexpressed in corneal epithelial cell layer with corneal epithelial cell marker CK12 (red fluorescence), which indicated galectin-3 was located in corneal epithelium. Moreover, galectin-3 and neutrophil marker CD11b were coexpressed in corneal epithelial cells and stroma layer, suggesting galectin-3 and neutrophils were correlated in immune responses of the fungal keratitis (Figure 3(b)). It was implied that the expression of galectin-3 has a positive effect on neutrophil recruitment in fungal keratitis.

### 3.4. Galectin-3 Knockout Significantly Affected the Progression of Fungal Keratitis

**Figure 4:**
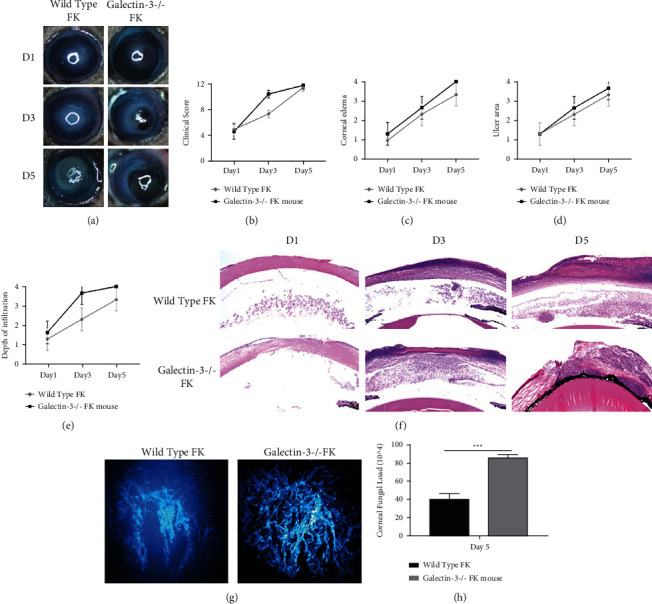
The model of fungal keratitis was established in galectin-3 knockout mice and wild type mice, and anterior segment imaging (a), clinical score (b–d), HE staining (e), two-photon microscopy (TPM) observation (g), and corneal fungal load (CFU) (h) detection were performed. The condition of galectin-3^−/−^ mice deteriorated rapidly on the third day after infection compared with the control group. Meanwhile, the number of fungal mycelia in corneas of C57 mice was much more than that of galectin-3^−/−^ mice. Magnification: photographic 16 times, HE 100 times. ^∗^*p* < 0.05;  ^∗∗^*p* < 0.01;  ^∗∗∗^*p* < 0.001; NS: no statistical difference.

To further study the regulation of galectin-3 in fungal keratitis, we established the mouse model of the wild type mice and galectin-3 knockout (galectin-3^−/−^) mice to evaluate the effect on the course of the disease. Anterior segment photography (Figure 4(a)) and clinical scores (galectin-3^−/−^ FK vs. wild type FK D1: 5.60 ± 0.15 vs. 5.50 ± 0.10; D3: 8.70 ± 0.20 vs. 10.60 ± 0.25; D5: 11.50 ± 0.20 vs. 11.90 ± 0.10) (Figure 4(b)) showed that compared with the wild type FK group, the condition of galectin-3^−/−^ FK group aggravated rapidly on the third day after infection, mainly caused by increased perforation rate (galectin-3^−/−^ FK vs. wild type FK D1: 1.33 ± 0.58 vs. 1.67 ± 0.58; D3: 2.33 ± 0.58 vs. 3.67 ± 0.58; D5: 3.33 ± 0.58 vs. 4.00 ± 0.00) (Figure 4(e)). While the trend of corneal edema (galectin-3^−/−^ FK vs. wild type FK D1: 1.00 ± 0.00 vs. 1.33 ± 0.58; D3: 2.33 ± 0.58 vs. 2.67 ± 0.58; D5: 3.33 ± 0.58 vs. 4.00 ± 0.00) and ulcer coverage (galectin-3^−/−^ FK vs. wild type FK D1: 1.33 ± 0.58 vs. 1.33 ± 0.58; D3: 2.33 ± 0.58 vs. 3.67 ± 0.57; D5: 3.33 ± 0.58 vs. 3.67 ± 0.58) was similar between the two groups (Figures 4(c) and 4(d)). It implied that the antifunal immune function of inflammatory cells to fight off fungal attacks was damaged by galectin-3 knockout. Pathological sections also showed that the are of inflammation and corneal edema severity in the mouse model of galectin-3^−/−^ fungal keratitis group were significantly increased than those in wild type FK group, and the depth of inflammatory cell infiltration gradually increased (Figure 4(f)), which was consistent with the disease scores.

Moreover, the effects of galectin-3 on anti-fungal activity were verified in this study. Two-photon microscopy was used to observe the number and morphology of fungal mycelia in corneas of wild type FK mice and galectin-3^−/−^ FK mice on day 5 after infection. It was found that the mycelium in the corneas of galectin-3^−/−^ FK mice was significantly denser than that in the normal mice (Figure 4(g)). Corresponding to this, the results of corneal fungal load (CFU) (Figure 4(h)) showed that on day 5 after infection, the CFU of wild type FK mice and galectin-3^−/−^ FK mice was 45.00 ± 5.78∗10^4^ and 90.67 ± 2.19 (P < 0.01), which mean the fungi in the galectin-3^−/−^ model grew well and vigorously. The results above suggested that galectin-3 may prevent and/or inhibit the fungal keratitis progression and positively regulate the fungal clearance of inflammatory cells.

### 3.5. Galectin-3 Knockout Impaired the Recruitment of Neutrophils in the Mouse Model of Fungal Keratitis

**Figure 5:**
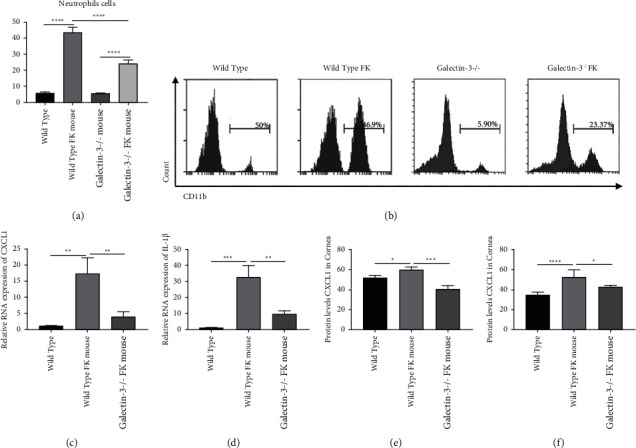
The levels of the activation of neutrophils in corneas (a, b) and the expression levels of CXCL1 (c, e, f) and IL-1*β* (d) in normal C57 mice, C57 model, galectin-3^−/−^wild type mice, and galectin-3^−/−^ model were detected. The deletion of galectin-3 significantly inhibited the neutrophil activation and the expression of the related chemokines CXCL1 and IL-1*β*. ^∗^*p* < 0.05;  ^∗∗^*p* < 0.01;  ^∗∗∗^*p* < 0.001; NS: no statistical difference.

To illustrate the effect of galectin-3 on immune cells in the mouse model of fungal keratitis, we first examined neutrophils' levels under different conditions. Flow cytometry analysis showed that neutrophils (CD45+ ly6G+ CD11b+) were significantly activated in corneas in the normal mice with fungal keratitis. At the same time, this phenomenon was inhibited in galectin-3^−/−^ disease model (wild type vs. wild type model: 5.78 ± 0.39 vs.43.57 ± 1.83%, *p* < 0.001; wild type model vs. galectin-3^−/−^ model: 5.69 ± 0.13 vs.24.19 ± 1.28%, *p* < 0.001) (Figures 5(a) and 5(b)).

In addition, corneas of mice were collected 5 days after infection to detect the RNA and protein levels of neutrophil chemokine CXCL1 and IL-1*β*. The results showed that the RNA levels of CXCL1 were increased in normal mice and decreased in knockout mice (wild type vs. wild type FK: 1.07 ± 0.07 vs.17.27 ± 2.86, *p* < 0.01; wild type FK vs. galectin-3^−/−^ FK: 17.27 ± 2.86 vs.3.87 ± 0.87, *p* < 0.05) (Figure 5(c)), and the protein levels in the corneas showed the same trend (wild type vs. wild type FK: 51.56 ± 1.24 vs.59.98 ± 1.03 ng/mL, *p* < 0.01; wild type vs. galectin-3^−/−^ FK: 59.98 ± 1.03 vs.40.27 ± 1.98, *p* < 0.001) (Figure 5(e)) as well as in the lymph nodes (wild type vs. wild type FK: 34.11 ± 3.13 vs.51.79 ± 7.74 ng/mL, *p* < 0.001; wild type FK vs. galectin-3^−/−^ FK: 51.79 ± 7.74 vs.42.25 ± 1.92, *p* < 0.05) (Figure 5(f)). The expression trend of IL-1*β* in the levels of protein (wild type vs. wild type FK: 1.07 ± 0.12 vs.32.53 ± 7.47, *p* < 0.01; wild type FK vs. galectin-3^−/−^ FK: 32.53 ± 7.47 vs.9.53 ± 1.78, *p* < 0.01) (Figure 5(d)) was similar to CXCL1 with statistical differences. These data above implied that galectin-3 played an immune role by promoting the activation of neutrophils in fungal keratitis. Furthermore, decreased activity of neutrophils, which are responsible for fungus control and antigen presentation, also explains the faster corneal perforation in the galectin-3^−/−^ mice of fungal keratitis.

## 4. Discussion

Galectin-3 is a master regulator of infectious disease. This study demonstrated that galectin-3 might play an important antifungal role in immunological responses to fight against fungal invasion by neutrophil recruitment in the FK mouse model. It was verified that galectin-3 knockout mice suffered a much more severe onset of FK with higher clinical scores of corneal lesion depth, area, and corneal edema degree compared to the wild-type mice. Lower levels of neutrophils and pro-inflammatory cytokines were found in the galectin-3 knockout mice group compared to the wild-type mouse model group, which indicates the regulatory role of galectin-3 in immune responses in vivo. The fungal load and fungal morphological activity were much higher in the galectin-3 knockout group than in the model group as well. These data suggested that galectin-3 might be a necessary regulatory biomarker in immunological responses to fight off fungal invasion on the ocular surface, and galectin-3 knockout could impair the antifungal immune response to a great extent.

Galectin-3 plays a pivotal role in microbial infection, such as bacteria, fungi, viruses, and parasites [32–34]. The mainstream points of view on galectin-3 in infectious disease pathogenesis support it to be beneficial. For example, In virus infectious diseases, galectin-3 tended to affect viruses such as HIV-1 (human immunodeficiency virus-1), MV-Mp (Minute virus of mouse protoparvovirus), and EV71 (Enterovirus 71), such as viral combination, replication, budding, and transmission, followed by infection-associated inflammation, and endogenous galectins as cellular factors by regulating viral infection interacting with cellular components under viral hijacking or inflammation led by virus replication [35]. Galectin-3 was recognized as a target to regulate viral immune response to modulate virus infection. In bacterial infection, the majority of researches on bacterial infection showed that streptococcus in galectin-3 knockout mice had a higher bacterial burden and enhanced nephritis and renal dysfunction in comparison with wild-type mice [36, 37]. Many studies demonstrated that galectin-3 appears to mediate neutrophil migration and recruitment in bacteria-caused infection animal models [37]. Our study was in agreement that galectin-3 knockout is detrimental to infectious disease recovery, leading to severe disease development and even corneal perforation. It is assumed that galectin-3 might fulfil its immunological regulation by recruiting key immune cells subtypes of FK pathogenesis, especially neutrophil recruitment.

Galectin-3 is important for both the activation and recruitment of neutrophils in the immune response of infectious diseases [20]. Neutrophils reacted positively in response to fungi invasion, killing and clearing foreign pathogens through phagocytosis. Studies on the galectin-3 receptors on neutrophils reported that adhesion between the neutrophils and inherent endothelial cells increased, so did the expression level of proinflammatory cytokines [37]. The recruitment and activation effect of galectin-3 on neutrophils demonstrated that inhibition of galectin-3 could be the reason of the down-regulation of innate immune responses in inflammatory disorders, especially infectious diseases. Recent studies showed that galectin-3 knockout mice show an elevated number of neutrophils in the primary focus of infection and reduced fungal loads in the fungal pneumonia lesions and peritoneal cavity caused by fungal infection [30]. Protective effects of galectin-3 deficiency in fungal keratitis proved the regulatory role of galectin-3 of neutrophil cell subtypes; thereby, our data hints at the importance of galectin-3 for innate immunity in fungal infection. Together, the current study supported the regulatory role of galectin-3 in the activation and recruitment of neutrophils in inflammatory diseases, especially infectious diseases. Our study proved the regulatory role of galectin-3 for key immune subtypes in antifungal inflammation. Speaking of galectin-3 involved immune-mediated cytokines, we also testified downstream cytokines in the inflammatory cascade; the expression levels of protein like CXCL1 and IL-1*β* were increased apparently. These are positively correlated with neutrophils in inflammatory responses. CXCL1 is an upstream signaling site for active migration of monocytes and neutrophils to gather timely in corneal lesion tissue. IL-1*β* contributes to the inflammatory cascades under the regulation of NLRP3 inflammasome, a hub protein complex that senses and cleaves cytokines into functioning forms.

Once the innate immune system recognizes pathogens, the barrier of the ocular surface will be the first line force to fight against fungal invasion. The ocular surface barrier consists of corneal epithelial cells, conjunctival cells, and so on [29]. Galectin-3 promotes barrier function at the vessel mucosa, skin layer, and ocular surface to protect the internal system from interaction to microbe everywhere. Galectin-3 is reported to be effective in controlling infection extension in bacterial lung infection and renal fungi infection [37, 38]. The barrier function of HCE in the human ocular surface contributes to the healthy microenvironment to protect inner cells from detrimental factors like oxidative stress, physical trauma and overstressed vision working load. It is assumed that ocular barrier function might be important to keep pathogens from settlement and generation [39, 40]. Our data showed that the knockout of galectin-3 intensifies the ocular barrier's damage in the fungal invasion. The lack of galectin-3 might do evil in fungal clearance, for the better growing status in corneal tissue under the observation of a two-photon microscope. Our data suggested that galectin-3 might regulate the barrier function to prevent tissue damage from the beginning of the inflammatory disease in fungal infectious disease, which indicated the key role of galectin-3 in infectious disease in a larger field.

In our study, it was shown that galectin-3 inhibition decreased neutrophils in the FK mouse model. The antifungal immune response occurs due to tissue infection and/or barrier function damage. Antifungal immune response aims at removing the invaders and rebalance local homeostasis. Galectin-3 regulates the anti-infection immune signaling pathway in fungal keratits. It is a process that is crucial to the host immune system in which neutrophils are activated and recruited. Therefore, targeting galectin-3 in anti-infection immune response would be a potential therapeutic alternative for fungal keratitis. With a better understanding of the molecular and cellular mechanism of galectin-3, it is pertinent to provide a new antifungal therapy for patients with fungal keratitis, and/or patients suffering from other fungal infectious diseases.

## Data Availability

The data and materials used to support the findings of this study are available from the corresponding author upon request.
